# Exploring healthcare provider’s views on potential causes and solutions of waiting lists in mental health services in Ireland

**DOI:** 10.1192/j.eurpsy.2025.2164

**Published:** 2025-08-26

**Authors:** J. Khan, M. Whitty, M. Nadeem

**Affiliations:** 1 Psychiatry, Connolly Hospital Blanchardstown Dublin, Dublin; 2 Psychiatry, South Meath Mental Health service, Ashbourne, Ireland

## Abstract

**Introduction:**

This study focused on one of the key components of mental healthcare provision: waiting lists in the community mental health service in County Louth and Meath, Ireland. The background context addresses the increasing demand for healthcare, shortage of resources, administrative hindrances, and the impact of waiting time on patient outcomes and satisfaction.

**Objectives:**

The objectives of the research were to Identify the primary causes of waiting lists in the community mental health services of County Louth and Meath in healthcare provider’s opinions and their views on potential solutions to reduce waiting lists and improve service delivery.

**Methods:**

A mixed-methods approach was applied to conduct a comprehensive assessment of waiting list causes and potential solutions based on the combination of qualitative and quantitative methods. The main qualitative methods encompassed semi-structured interviews with healthcare professionals and thematic analysis of their perceptions. The quantitative part of the study consisted of a survey distributed among members of the multidisciplinary mental health teams to collect information regarding perceptions, challenges, and potential solutions to the waiting list problems.

**Results:**

The study showed a variety of issues in the human factor of waiting lists, including insufficient staffing, high demand for services, administrative delays, limited resources, and communication failures between mental health and primary care teams. The mental healthcare professionals in County Louth and Meath voiced their apprehensions concerning patients’ wait times, resource scarcity, and the effect of these problems on the performance of services and patient outcomes. The general findings uncovered that 89.3% of respondents recognized insufficient staffing as the essential challenge in waiting list management, with 82.1% likewise featuring the higher demand for services as a significant variable adding to waiting lists.

**Conclusions:**

Carefully assessing these issues is vital, and evidence-based steps are to be employed in managing the waiting lists in the community mental health services in County Louth and Meath, Ireland. Suggestions on increased staffing, resource investment, technology leveraging, process optimization, and enhanced communication between primary care and psychiatry are the essence of potential solutions. Undertaking these suggestions will enable mental health services to increase efficiency, provide a better patient experience, and subsequently improve overall service delivery while reducing the consequences of long waiting times.

**Disclosure of Interest:**

None Declared

